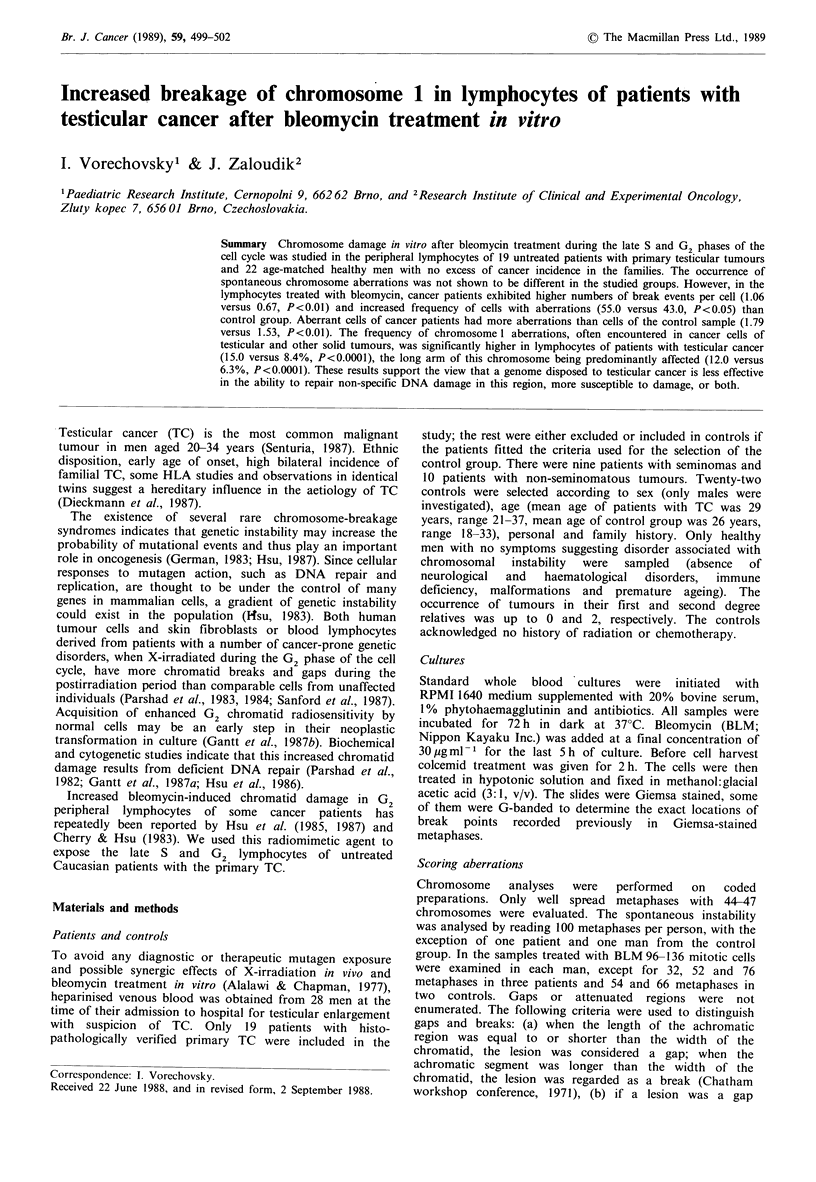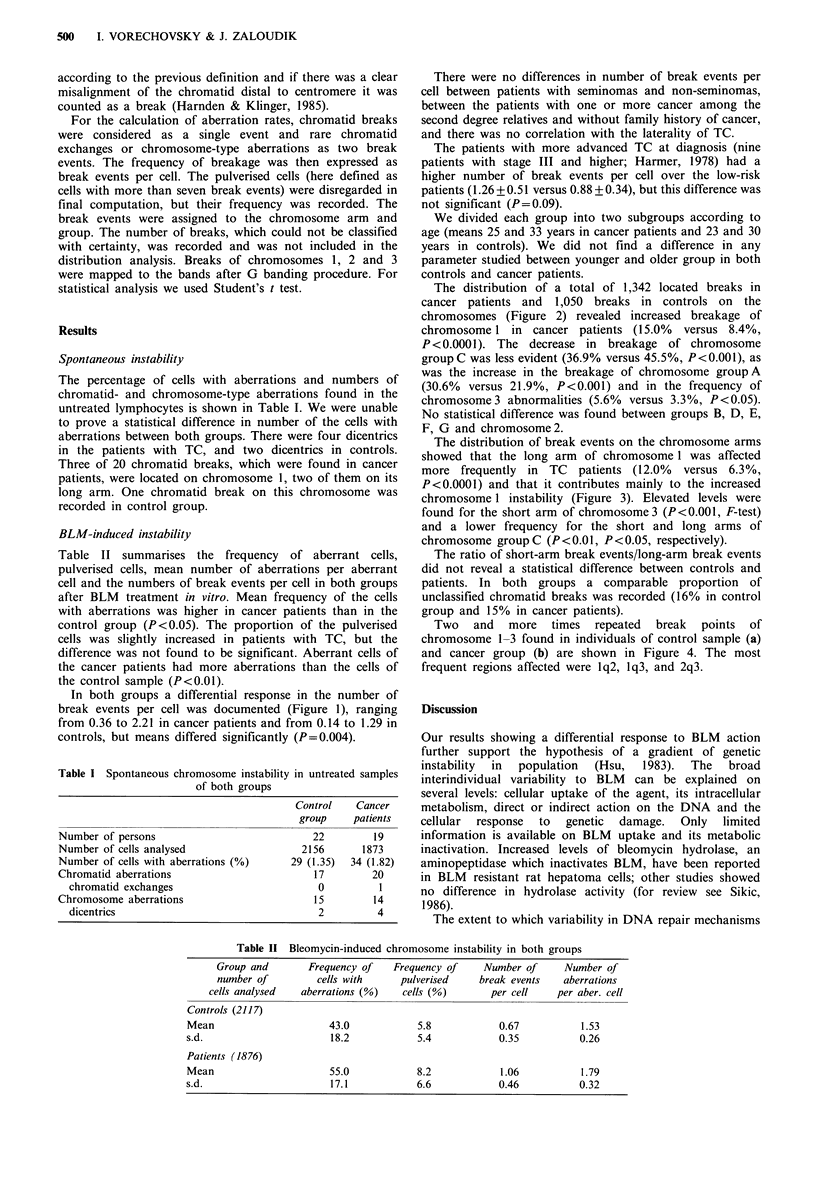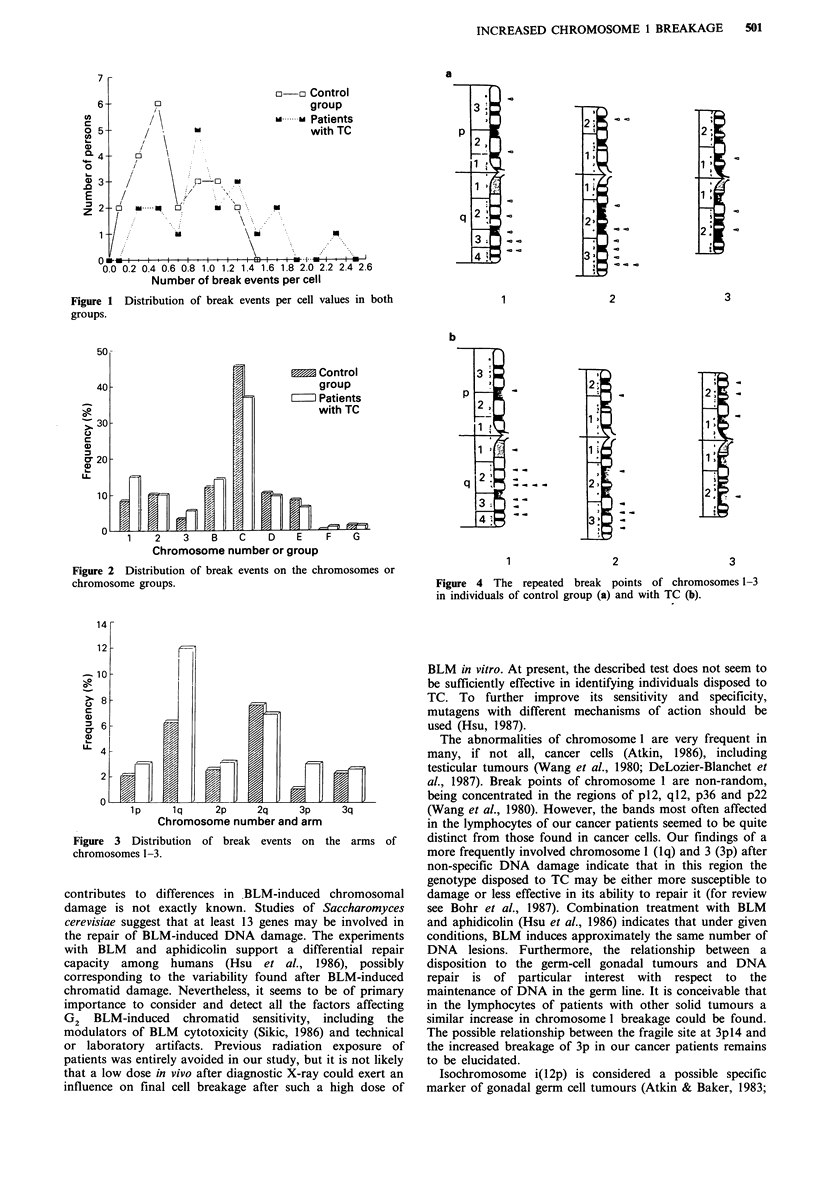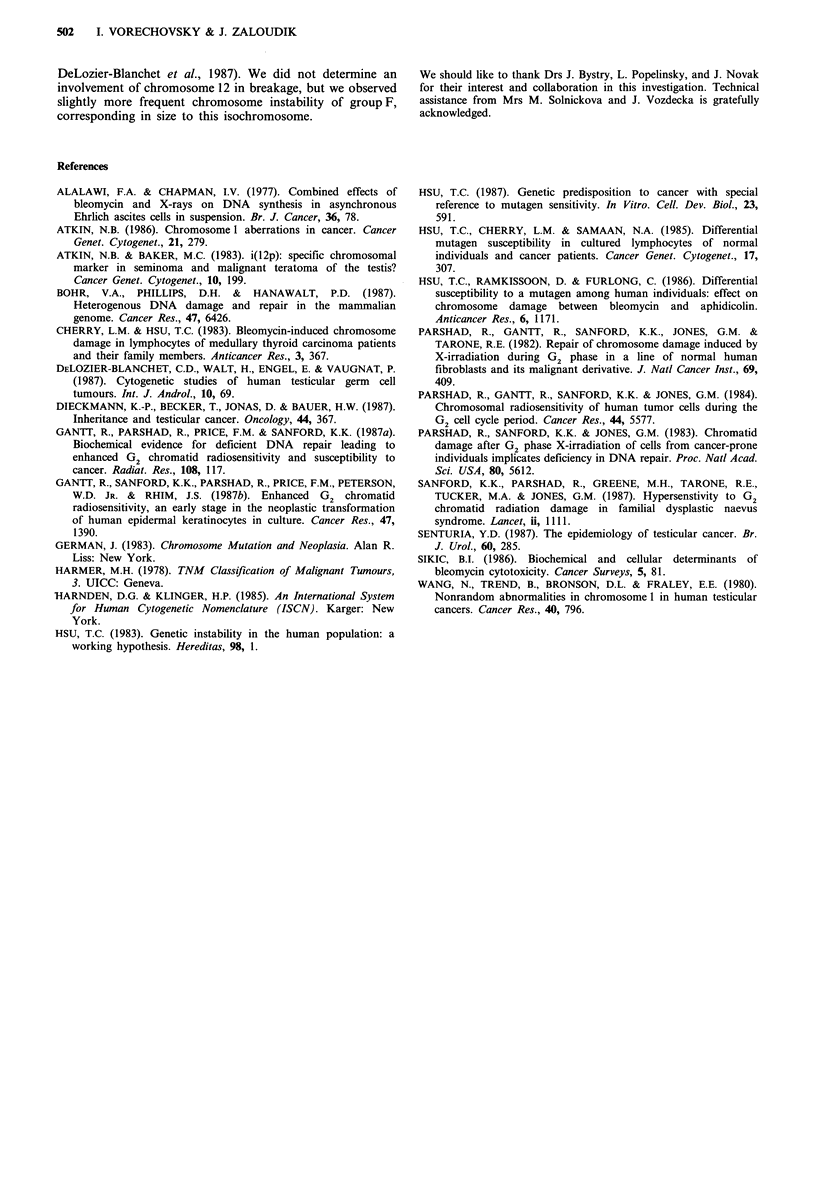# Increased breakage of chromosome 1 in lymphocytes of patients with testicular cancer after bleomycin treatment in vitro.

**DOI:** 10.1038/bjc.1989.103

**Published:** 1989-04

**Authors:** I. Vorechovsky, J. Zaloudik

**Affiliations:** Paediatric Research Institute, Brno, Czechoslovakia.

## Abstract

Chromosome damage in vitro after bleomycin treatment during the late S and G2 phases of the cell cycle was studied in the peripheral lymphocytes of 19 untreated patients with primary testicular tumours and 22 age-matched healthy men with no excess of cancer incidence in the families. The occurrence of spontaneous chromosome aberrations was not shown to be different in the studied groups. However, in the lymphocytes treated with bleomycin, cancer patients exhibited higher numbers of break events per cell (1.06 versus 0.67, P less than 0.01) and increased frequency of cells with aberrations (55.0 versus 43.0, P less than 0.05) than control group. Aberrant cells of cancer patients had more aberrations than cells of the control sample (1.79 versus 1.53, P less than 0.01). The frequency of chromosome 1 aberrations, often encountered in cancer cells of testicular and other solid tumours, was significantly higher in lymphocytes of patients with testicular cancer (15.0 versus 8.4%, P less than 0.0001), the long arm of this chromosome being predominantly affected (12.0 versus 6.3%, P less than 0.0001). These results support the view that a genome disposed to testicular cancer is less effective in the ability to repair non-specific DNA damage in this region, more susceptible to damage, or both.


Br. J. Cancer (1989), 59, 499–502 © The Macmillan Press Ltd., 1989

### Increased breakage of chromosome 1 in lymphocytes of patients with testicular cancer after bleomycin treatment *in vitro*

I. Vorechovsky[1] & J. Zaloudik[2]

[1]*Paediatric Research Institute, Cernopolni 9, 66262 Brno, and* [2]*Research Institute of Clinical and Experimental Oncology, Zluty kopec 7, 65601 Brno, Czechoslovakia.*

**Summary** Chromosome damage *in vitro* after bleomycin treatment during the late S and $G_2$ phases of the cell cycle was studied in the peripheral lymphocytes of 19 untreated patients with primary testicular tumours and 22 age-matched healthy men with no excess of cancer incidence in the families. The occurrence of spontaneous chromosome aberrations was not shown to be different in the studied groups. However, in the lymphocytes treated with bleomycin, cancer patients exhibited higher numbers of break events per cell (1.06 versus 0.67, $P<0.01$) and increased frequency of cells with aberrations (55.0 versus 43.0, $P<0.05$) than control group. Aberrant cells of cancer patients had more aberrations than cells of the control sample (1.79 versus 1.53, $P<0.01$). The frequency of chromosome 1 aberrations, often encountered in cancer cells of testicular and other solid tumours, was significantly higher in lymphocytes of patients with testicular cancer (15.0 versus 8.4%, $P<0.0001$), the long arm of this chromosome being predominantly affected (12.0 versus 6.3%, $P<0.0001$). These results support the view that a genome disposed to testicular cancer is less effective in the ability to repair non-specific DNA damage in this region, more susceptible to damage, or both.

Testicular cancer (TC) is the most common malignant tumour in men aged 20–34 years (Senturia, 1987). Ethnic disposition, early age of onset, high bilateral incidence of familial TC, some HLA studies and observations in identical twins suggest a hereditary influence in the aetiology of TC (Dieckmann *et al.*, 1987).

The existence of several rare chromosome-breakage syndromes indicates that genetic instability may increase the probability of mutational events and thus play an important role in oncogenesis (German, 1983; Hsu, 1987). Since cellular responses to mutagen action, such as DNA repair and replication, are thought to be under the control of many genes in mammalian cells, a gradient of genetic instability could exist in the population (Hsu, 1983). Both human tumour cells and skin fibroblasts or blood lymphocytes derived from patients with a number of cancer-prone genetic disorders, when X-irradiated during the $G_2$ phase of the cell cycle, have more chromatid breaks and gaps during the postirradiation period than comparable cells from unaffected individuals (Parshad *et al.*, 1983, 1984; Sanford *et al.*, 1987). Acquisition of enhanced $G_2$ chromatid radiosensitivity by normal cells may be an early step in their neoplastic transformation in culture (Gantt *et al.*, 1987*b*). Biochemical and cytogenetic studies indicate that this increased chromatid damage results from deficient DNA repair (Parshad *et al.*, 1982; Gantt *et al.*, 1987*a*; Hsu *et al.*, 1986).

Increased bleomycin-induced chromatid damage in $G_2$ peripheral lymphocytes of some cancer patients has repeatedly been reported by Hsu *et al.* (1985, 1987) and Cherry & Hsu (1983). We used this radiomimetic agent to expose the late S and $G_2$ lymphocytes of untreated Caucasian patients with the primary TC.

### Materials and methods

### *Patients and controls*

To avoid any diagnostic or therapeutic mutagen exposure and possible synergic effects of X-irradiation *in vivo* and bleomycin treatment *in vitro* (Alalawi & Chapman, 1977), heparinised venous blood was obtained from 28 men at the time of their admission to hospital for testicular enlargement with suspicion of TC. Only 19 patients with histopathologically verified primary TC were included in the study; the rest were either excluded or included in controls if the patients fitted the criteria used for the selection of the control group. There were nine patients with seminomas and 10 patients with non-seminomatous tumours. Twenty-two controls were selected according to sex (only males were investigated), age (mean age of patients with TC was 29 years, range 21–37, mean age of control group was 26 years, range 18–33), personal and family history. Only healthy men with no symptoms suggesting disorder associated with chromosomal instability were sampled (absence of neurological and haematological disorders, immune deficiency, malformations and premature ageing). The occurrence of tumours in their first and second degree relatives was up to 0 and 2, respectively. The controls acknowledged no history of radiation or chemotherapy.

### *Cultures*

Standard whole blood cultures were initiated with RPMI 1640 medium supplemented with 20% bovine serum, 1% phytohaemagglutinin and antibiotics. All samples were incubated for 72 h in dark at 37°C. Bleomycin (BLM; Nippon Kayaku Inc.) was added at a final concentration of 30 $\mu g\,ml^{-1}$ for the last 5 h of culture. Before cell harvest colcemid treatment was given for 2 h. The cells were then treated in hypotonic solution and fixed in methanol:glacial acetic acid (3:1, v/v). The slides were Giemsa stained, some of them were G-banded to determine the exact locations of break points recorded previously in Giemsa-stained metaphases.

### *Scoring aberrations*

Chromosome analyses were performed on coded preparations. Only well spread metaphases with 44–47 chromosomes were evaluated. The spontaneous instability was analysed by reading 100 metaphases per person, with the exception of one patient and one man from the control group. In the samples treated with BLM 96–136 mitotic cells were examined in each man, except for 32, 52 and 76 metaphases in three patients and 54 and 66 metaphases in two controls. Gaps or attenuated regions were not enumerated. The following criteria were used to distinguish gaps and breaks: (a) when the length of the achromatic region was equal to or shorter than the width of the chromatid, the lesion was considered a gap; when the achromatic segment was longer than the width of the chromatid, the lesion was regarded as a break (Chatham workshop conference, 1971), (b) if a lesion was a gap

Correspondence: I. Vorechovsky.
Received 22 June 1988, and in revised form, 2 September 1988.

according to the previous definition and if there was a clear misalignment of the chromatid distal to centromere it was counted as a break (Harnden & Klinger, 1985).

For the calculation of aberration rates, chromatid breaks were considered as a single event and rare chromatid exchanges or chromosome-type aberrations as two break events. The frequency of breakage was then expressed as break events per cell. The pulverised cells (here defined as cells with more than seven break events) were disregarded in final computation, but their frequency was recorded. The break events were assigned to the chromosome arm and group. The number of breaks, which could not be classified with certainty, was recorded and was not included in the distribution analysis. Breaks of chromosomes 1, 2 and 3 were mapped to the bands after G banding procedure. For statistical analysis we used Student's $t$ test.

### Results

### *Spontaneous instability*

The percentage of cells with aberrations and numbers of chromatid- and chromosome-type aberrations found in the untreated lymphocytes is shown in Table I. We were unable to prove a statistical difference in number of the cells with aberrations between both groups. There were four dicentrics in the patients with TC, and two dicentrics in controls. Three of 20 chromatid breaks, which were found in cancer patients, were located on chromosome 1, two of them on its long arm. One chromatid break on this chromosome was recorded in control group.

### *BLM-induced instability*

Table II summarises the frequency of aberrant cells, pulverised cells, mean number of aberrations per aberrant cell and the numbers of break events per cell in both groups after BLM treatment *in vitro*. Mean frequency of the cells with aberrations was higher in cancer patients than in the control group ($P<0.05$). The proportion of the pulverised cells was slightly increased in patients with TC, but the difference was not found to be significant. Aberrant cells of the cancer patients had more aberrations than the cells of the control sample ($P<0.01$).

In both groups a differential response in the number of break events per cell was documented (Figure 1), ranging from 0.36 to 2.21 in cancer patients and from 0.14 to 1.29 in controls, but means differed significantly ($P=0.004$).

**Table I** Spontaneous chromosome instability in untreated samples of both groups

| | *Control group* | *Cancer patients* |
|---|---|---|
| Number of persons | 22 | 19 |
| Number of cells analysed | 2156 | 1873 |
| Number of cells with aberrations (%) | 29 (1.35) | 34 (1.82) |
| Chromatid aberrations | 17 | 20 |
| chromatid exchanges | 0 | 1 |
| Chromosome aberrations | 15 | 14 |
| dicentrics | 2 | 4 |

There were no differences in number of break events per cell between patients with seminomas and non-seminomas, between the patients with one or more cancer among the second degree relatives and without family history of cancer, and there was no correlation with the laterality of TC.

The patients with more advanced TC at diagnosis (nine patients with stage III and higher; Harmer, 1978) had a higher number of break events per cell over the low-risk patients ($1.26 \pm 0.51$ versus $0.88 \pm 0.34$), but this difference was not significant ($P=0.09$).

We divided each group into two subgroups according to age (means 25 and 33 years in cancer patients and 23 and 30 years in controls). We did not find a difference in any parameter studied between younger and older group in both controls and cancer patients.

The distribution of a total of 1,342 located breaks in cancer patients and 1,050 breaks in controls on the chromosomes (Figure 2) revealed increased breakage of chromosome 1 in cancer patients (15.0% versus 8.4%, $P<0.0001$). The decrease in breakage of chromosome group C was less evident (36.9% versus 45.5%, $P<0.001$), as was the increase in the breakage of chromosome group A (30.6% versus 21.9%, $P<0.001$) and in the frequency of chromosome 3 abnormalities (5.6% versus 3.3%, $P<0.05$). No statistical difference was found between groups B, D, E, F, G and chromosome 2.

The distribution of break events on the chromosome arms showed that the long arm of chromosome 1 was affected more frequently in TC patients (12.0% versus 6.3%, $P<0.0001$) and that it contributes mainly to the increased chromosome 1 instability (Figure 3). Elevated levels were found for the short arm of chromosome 3 ($P<0.001$, $F$-test) and a lower frequency for the short and long arms of chromosome group C ($P<0.01$, $P<0.05$, respectively).

The ratio of short-arm break events/long-arm break events did not reveal a statistical difference between controls and patients. In both groups a comparable proportion of unclassified chromatid breaks was recorded (16% in control group and 15% in cancer patients).

Two and more times repeated break points of chromosome 1–3 found in individuals of control sample (**a**) and cancer group (**b**) are shown in Figure 4. The most frequent regions affected were 1q2, 1q3, and 2q3.

### Discussion

Our results showing a differential response to BLM action further support the hypothesis of a gradient of genetic instability in population (Hsu, 1983). The broad interindividual variability to BLM can be explained on several levels: cellular uptake of the agent, its intracellular metabolism, direct or indirect action on the DNA and the cellular response to genetic damage. Only limited information is available on BLM uptake and its metabolic inactivation. Increased levels of bleomycin hydrolase, an aminopeptidase which inactivates BLM, have been reported in BLM resistant rat hepatoma cells; other studies showed no difference in hydrolase activity (for review see Sikic, 1986).

The extent to which variability in DNA repair mechanisms

**Table II** Bleomycin-induced chromosome instability in both groups

| *Group and number of cells analysed* | *Frequency of cells with aberrations (%)* | *Frequency of pulverised cells (%)* | *Number of break events per cell* | *Number of aberrations per aber. cell* |
|---|---|---|---|---|
| *Controls (2117)* | | | | |
| Mean | 43.0 | 5.8 | 0.67 | 1.53 |
| s.d. | 18.2 | 5.4 | 0.35 | 0.26 |
| *Patients (1876)* | | | | |
| Mean | 55.0 | 8.2 | 1.06 | 1.79 |
| s.d. | 17.1 | 6.6 | 0.46 | 0.32 |

**Figure 1** Distribution of break events per cell values in both groups.

**Figure 2** Distribution of break events on the chromosomes or chromosome groups.

**Figure 3** Distribution of break events on the arms of chromosomes 1–3.

**Figure 4** The repeated break points of chromosomes 1–3 in individuals of control group (**a**) and with TC (**b**).

contributes to differences in BLM-induced chromosomal damage is not exactly known. Studies of *Saccharomyces cerevisiae* suggest that at least 13 genes may be involved in the repair of BLM-induced DNA damage. The experiments with BLM and aphidicolin support a differential repair capacity among humans (Hsu *et al.*, 1986), possibly corresponding to the variability found after BLM-induced chromatid damage. Nevertheless, it seems to be of primary importance to consider and detect all the factors affecting $G_2$ BLM-induced chromatid sensitivity, including the modulators of BLM cytotoxicity (Sikic, 1986) and technical or laboratory artifacts. Previous radiation exposure of patients was entirely avoided in our study, but it is not likely that a low dose *in vivo* after diagnostic X-ray could exert an influence on final cell breakage after such a high dose of BLM *in vitro*. At present, the described test does not seem to be sufficiently effective in identifying individuals disposed to TC. To further improve its sensitivity and specificity, mutagens with different mechanisms of action should be used (Hsu, 1987).

The abnormalities of chromosome 1 are very frequent in many, if not all, cancer cells (Atkin, 1986), including testicular tumours (Wang *et al.*, 1980; DeLozier-Blanchet *et al.*, 1987). Break points of chromosome 1 are non-random, being concentrated in the regions of p12, q12, p36 and p22 (Wang *et al.*, 1980). However, the bands most often affected in the lymphocytes of our cancer patients seemed to be quite distinct from those found in cancer cells. Our findings of a more frequently involved chromosome 1 (1q) and 3 (3p) after non-specific DNA damage indicate that in this region the genotype disposed to TC may be either more susceptible to damage or less effective in its ability to repair it (for review see Bohr *et al.*, 1987). Combination treatment with BLM and aphidicolin (Hsu *et al.*, 1986) indicates that under given conditions, BLM induces approximately the same number of DNA lesions. Furthermore, the relationship between a disposition to the germ-cell gonadal tumours and DNA repair is of particular interest with respect to the maintenance of DNA in the germ line. It is conceivable that in the lymphocytes of patients with other solid tumours a similar increase in chromosome 1 breakage could be found. The possible relationship between the fragile site at 3p14 and the increased breakage of 3p in our cancer patients remains to be elucidated.

Isochromosome i(12p) is considered a possible specific marker of gonadal germ cell tumours (Atkin & Baker, 1983;

DeLozier-Blanchet *et al.*, 1987). We did not determine an involvement of chromosome 12 in breakage, but we observed slightly more frequent chromosome instability of group F, corresponding in size to this isochromosome.

We should like to thank Drs J. Bystry, L. Popelinsky, and J. Novak for their interest and collaboration in this investigation. Technical assistance from Mrs M. Solnickova and J. Vozdecka is gratefully acknowledged.

### References

ALALAWI, F.A. & CHAPMAN, I.V. (1977). Combined effects of bleomycin and X-rays on DNA synthesis in asynchronous Ehrlich ascites cells in suspension. *Br. J. Cancer*, **36**, 78.

ATKIN, N.B. (1986). Chromosome 1 aberrations in cancer. *Cancer Genet. Cytogenet.*, **21**, 279.

ATKIN, N.B. & BAKER, M.C. (1983). i(12p): specific chromosomal marker in seminoma and malignant teratoma of the testis? *Cancer Genet. Cytogenet.*, **10**, 199.

BOHR, V.A., PHILLIPS, D.H. & HANAWALT, P.D. (1987). Heterogenous DNA damage and repair in the mammalian genome. *Cancer Res.*, **47**, 6426.

CHERRY, L.M. & HSU, T.C. (1983). Bleomycin-induced chromosome damage in lymphocytes of medullary thyroid carcinoma patients and their family members. *Anticancer Res.*, **3**, 367.

DELOZIER-BLANCHET, C.D., WALT, H., ENGEL, E. & VAUGNAT, P. (1987). Cytogenetic studies of human testicular germ cell tumours. *Int. J. Androl.*, **10**, 69.

DIECKMANN, K.-P., BECKER, T., JONAS, D. & BAUER, H.W. (1987). Inheritance and testicular cancer. *Oncology*, **44**, 367.

GANTT, R., PARSHAD, R., PRICE, F.M. & SANFORD, K.K. (1987*a*). Biochemical evidence for deficient DNA repair leading to enhanced $G_2$ chromatid radiosensitivity and susceptibility to cancer. *Radiat. Res.*, **108**, 117.

GANTT, R., SANFORD, K.K., PARSHAD, R., PRICE, F.M., PETERSON, W.D. JR. & RHIM, J.S. (1987*b*). Enhanced $G_2$ chromatid radiosensitivity, an early stage in the neoplastic transformation of human epidermal keratinocytes in culture. *Cancer Res.*, **47**, 1390.

GERMAN, J. (1983). *Chromosome Mutation and Neoplasia*. Alan R. Liss: New York.

HARMER, M.H. (1978). *TNM Classification of Malignant Tumours, 3*. UICC: Geneva.

HARNDEN, D.G. & KLINGER, H.P. (1985). *An International System for Human Cytogenetic Nomenclature (ISCN)*. Karger: New York.

HSU, T.C. (1983). Genetic instability in the human population: a working hypothesis. *Hereditas*, **98**, 1.

HSU, T.C. (1987). Genetic predisposition to cancer with special reference to mutagen sensitivity. *In Vitro. Cell. Dev. Biol.*, **23**, 591.

HSU, T.C., CHERRY, L.M. & SAMAAN, N.A. (1985). Differential mutagen susceptibility in cultured lymphocytes of normal individuals and cancer patients. *Cancer Genet. Cytogenet.*, **17**, 307.

HSU, T.C., RAMKISSOON, D. & FURLONG, C. (1986). Differential susceptibility to a mutagen among human individuals: effect on chromosome damage between bleomycin and aphidicolin. *Anticancer Res.*, **6**, 1171.

PARSHAD, R., GANTT, R., SANFORD, K.K., JONES, G.M. & TARONE, R.E. (1982). Repair of chromosome damage induced by X-irradiation during $G_2$ phase in a line of normal human fibroblasts and its malignant derivative. *J. Natl Cancer Inst.*, **69**, 409.

PARSHAD, R., GANTT, R., SANFORD, K.K. & JONES, G.M. (1984). Chromosomal radiosensitivity of human tumor cells during the $G_2$ cell cycle period. *Cancer Res.*, **44**, 5577.

PARSHAD, R., SANFORD, K.K. & JONES, G.M. (1983). Chromatid damage after $G_2$ phase X-irradiation of cells from cancer-prone individuals implicates deficiency in DNA repair. *Proc. Natl Acad. Sci. USA*, **80**, 5612.

SANFORD, K.K., PARSHAD, R., GREENE, M.H., TARONE, R.E., TUCKER, M.A. & JONES, G.M. (1987). Hypersenstivity to $G_2$ chromatid radiation damage in familial dysplastic naevus syndrome. *Lancet*, **ii**, 1111.

SENTURIA, Y.D. (1987). The epidemiology of testicular cancer. *Br. J. Urol.*, **60**, 285.

SIKIC, B.I. (1986). Biochemical and cellular determinants of bleomycin cytotoxicity. *Cancer Surveys*, **5**, 81.

WANG, N., TREND, B., BRONSON, D.L. & FRALEY, E.E. (1980). Nonrandom abnormalities in chromosome 1 in human testicular cancers. *Cancer Res.*, **40**, 796.